# Author Correction: Determinants of genome-wide distribution and evolution of uORFs in eukaryotes

**DOI:** 10.1038/s41467-021-22435-2

**Published:** 2021-03-31

**Authors:** Hong Zhang, Yirong Wang, Xinkai Wu, Xiaolu Tang, Changcheng Wu, Jian Lu

**Affiliations:** 1 State Key Laboratory of Protein and Plant Gene Research, Center for Bioinformatics, School of Life Sciences, Peking University, Beijing, China; 2 College of Biology, Hunan University, Changsha, China

**Keywords:** Molecular evolution, Population genetics, Functional genomics, Gene regulation

Correction to: *Nature Communications* 10.1038/s41467-021-21394-y, published online 17 February 2021.

The original version of this Article contained an error in the legend of Fig. 6, which incorrectly read ‘between species within the same taxa (green) or species in different taxa (red)’. The correct version states ‘between species within the same taxa (brown) or species in different taxa (green)’.

The original version of this Article also contained errors in the references to figures in the ‘Comparing the canonical versus noncanonical uORFs in repressing CDS translation in human populations’ section of the Results. In the second paragraph of this section, Fig. 6b was incorrectly referred to, this incorrectly read ‘A general trend was the slope values were overall positive for the canonical uORFs, while the slope values for the noncanonical uORFs fluctuated around 0 (Fig. 6b).’ The correct version states ‘A general trend was the slope values were overall positive for the canonical uORFs, while the slope values for the noncanonical uORFs fluctuated around 0 (Fig. 7b).’ In the third paragraph of this section, Fig. 6c and d were incorrectly referred to, this incorrectly read ‘Although occasionally the non-uORF allele had a stronger repressive effect than the uORF allele, the general trend was that the uORF allele had a stronger effect than the non-uORF allele in suppressing translation (Fig. 6c, d). Moreover, a significantly higher proportion of the canonical (55%, 23/42) than the noncanonical (26%, 10/38) uORFs exhibited the pattern that the annotated uORF allele showed a significantly stronger repressive effect on the CDS translation than the non-uORF allele (*P* = 0.013, Fisher’s exact test, Fig. 6c, d).’ The correct version states ‘Although occasionally the non-uORF allele had a stronger repressive effect than the uORF allele, the general trend was that the uORF allele had a stronger effect than the non-uORF allele in suppressing translation (Fig. 7c, d). Moreover, a significantly higher proportion of the canonical (55%, 23/42) than the noncanonical (26%, 10/38) uORFs exhibited the pattern that the annotated uORF allele showed a significantly stronger repressive effect on the CDS translation than the non-uORF allele (*P* = 0.013, Fisher’s exact test, Fig. 7c, d).’

These have been corrected in both the PDF and HTML versions of the Article.

