# Supplementary material for: Determinants of genome-wide distribution and evolution of uORFs in eukaryotes
Source: Nat Commun. 2021 Feb 17;12:1076. doi: 10.1038/s41467-021-21394-y (PMC7889888; doi:10.1038/s41467-021-21394-y)
Supplement: Supplementary file 1 — Supplementary Information [file 41467_2021_21394_MOESM1_ESM.pdf]

Supplementary Information

Determinants of genome-wide distribution and evolution of uORFs in eukaryotes

Hong Zhang, Yirong Wang, Xinkai Wu, Xiaolu Tang, Changcheng Wu and Jian Lu

Table of contents

Supplementary Tables 2

Supplementary Figures 5

Supplementary References 24

Supplementary Tables

Supplementary Table 1. The number of translated upstream open reading frames (uORFs) that are supported by ribosome-protected fragments (RPFs) in ribosome profiling (Ribo-Seq) and translation initiation site profiling datasets.

Species	Total number of unique uORFs	Supported by RPFs	Supported by initiating RPFs	Supported by both
Homo sapiens	78,003	54,121	26,344	25,902
Mus musculus	62,669	42,907	16,245	16,193
Rattus norvegicus	22,580	14,878	-	-
Danio rerio	80,493	42,173	-	-
Drosophila melanogaster	35,874	31,974	15,195	14,993
Arabidopsis thaliana	58,019	21,741	-	-
Saccharomyces cerevisiae	2,138	2,001	-	-

Supplementary Table 2. The effective population size (N_e) of 14 animals reported in previous studies.

Species	N_e	Study
Anser brachyrhynchus	6,550	Ruokonen et al., 2005, *Mol. Ecol.*[1]
Bos taurus	90,000	MacEachern et al., 2009, *BMC Genomics*[2]
Calidris pygmaea	438	Clark et al., 2018, *Oryx*[3]
Canis familiaris	2,000	Freedman et al., 2014, *PLOS Genet.*[4]
Carlito syrichta	15,000	Schmitz et al., 2016, *Nat. Commun.*[5]
Drosophila melanogaster	1,150,000	Gossmann et al., 2012, *Genome Biol. Evol.*[6]
Drosophila pseudoobscura	798,607	Gossmann et al., 2012, *Genome Biol. Evol.*[6]
Homo sapiens	20,974	Gossmann et al., 2012, *Genome Biol. Evol.*[6]
Macaca mulatta	52,350	Xue et al., 2016, *Genome Res.*[7]
Mus musculus	160,000	Salcedo et al., 2007, *Genetics*[8]
Pan paniscus	12,300	Yu et al., 2004, *Genetics*[9]
Pan troglodytes	21,300	Yu et al., 2004, *Genetics*[9]
Pongo abelii	26,800	Mailund et al., 2011, *PLOS Genet.*[10]
Rattus norvegicus	124,000	Deinum et al., 2015, *Mol. Biol. Evol.*[11]

Supplementary Table 3. Median values of the number of nonsynonymous changes per nonsynonymous site over the number of synonymous changes per synonymous site (ω) across all pairs of one-to-one orthologs in 37 pairs of closely-related species.

Species 1	Species 2	Median ω
Aedes aegypti	*Anopheles gambiae*	0.058
Aedes aegypti	*Culicoides sonorensis*	0.080
Anolis carolinensis	*Sphenodon punctatus*	0.097
Apis mellifera	*Bombus terrestris*	0.080
Apis mellifera	*Solenopsis invicta*	0.061
Apteryx owenii	*Dromaius novaehollandiae*	0.218
Bos taurus	*Capra hircus*	0.174
Bos taurus	*Sus scrofa*	0.135
Calidris pugnax	*Calidris pygmaea*	0.154
Calidris pugnax	*Melopsittacus undulatus*	0.144
Ciona savignyi	*Ciona intestinalis*	0.063
Danio rerio	*Astyanax mexicanus*	0.087
Danio rerio	*Ictalurus punctatus*	0.092
Drosophila melanogaster	*Drosophila pseudoobscura*	0.058
Drosophila melanogaster	*Drosophila simulans*	0.098
Drosophila melanogaster	*Drosophila yakuba*	0.090
Gallus gallus	*Anas platyrhynchos platyrhynchos*	0.130
Gallus gallus	*Anser brachyrhynchus*	0.126
Heliconius melpomene	*Melitaea cinxia*	0.052
Homo sapiens	*Callithrix jacchus*	0.183
Homo sapiens	*Macaca mulatta*	0.199
Homo sapiens	*Microcebus murinus*	0.147
Monodelphis domestica	*Phascolarctos cinereus*	0.154
Monodelphis domestica	*Sarcophilus harrisii*	0.148
Mus musculus	*Cricetulus griseus crigri*	0.145
Mus musculus	*Heterocephalus glaber male*	0.121
Mus musculus	*Oryctolagus cuniculus*	0.106
Mus musculus	*Rattus norvegicus*	0.139
Parus major	*Ficedula albicollis*	0.152
Parus major	*Manacus vitellinus*	0.152
Poecilia mexicana	*Gambusia affinis*	0.204
Poecilia mexicana	*Xiphophorus maculatus*	0.204
Seriola dumerili	*Mastacembelus armatus*	0.153
Seriola dumerili	*Scophthalmus maximus*	0.143
Tribolium castaneum	*Anoplophora glabripennis*	0.059
Tribolium castaneum	*Dendroctonus ponderosae*	0.068
Ursus americanus	*Mustela putorius furo*	0.144

Supplementary Table 4. The fraction of newly fixed upstream open reading frames (uORFs) driven by positive selection (α_{asym}) in highly-expressed genes or lowly-expressed genes in humans and flies.

Dataset		α_{asym} (95% confidence interval)	
		**Highly-expressed genes**	**Lowly-expressed genes**
Human (CpG to TpG mutations in 1000 Genomes)	Branch 1	0.83(0.28~1.00)	0.16(-0.30~0.62)
	Branch 2	0.86(0.24~1.00)	0.08(-0.51~0.66)
	Branch 3	0.86(0.14~1.00)	0.15(-0.30~0.59)
Fly (mutations in DGRP2)		0.38(0.22~0.49)	0.28(-1.00~0.39)

Supplementary Table 5. The ratios of observed over expected number (O/E ratio) of AUG triplets in 5' untranslated regions (UTRs) that are proximal or distal to coding sequences (CDSs) in the 5 protists that have significantly longer 5' UTRs than the other protists.

Cutoff of proximal 5' UTR regions	Species	O/E ratio (95% confidence interval)	
		**Proximal**	**Distal**
100 nt	*Cystoisospora suis*	0.865 (0.848~0.882)	1.208 (1.201~1.215)
	Nannochloropsis gaditana	0.683 (0.671~0.698)	1.143 (1.130~1.155)
	Plasmodium vivax	0.636 (0.613~0.661)	1.098 (1.079~1.116)
	Plasmodium yoelii	0.822 (0.803~0.843)	1.057 (1.048~1.067)
	Toxoplasma gondii	0.664 (0.648~0.682)	1.048 (1.039~1.056)
150 nt	*Cystoisospora suis*	0.926 (0.912~0.942)	1.215 (1.208~1.222)
	Nannochloropsis gaditana	0.793 (0.780~0.806)	1.157 (1.143~1.171)
	Plasmodium vivax	0.725 (0.704~0.748)	1.124 (1.105~1.144)
	Plasmodium yoelii	0.884 (0.866~0.902)	1.061 (1.052~1.071)
	Toxoplasma gondii	0.723 (0.708~0.739)	1.061 (1.052~1.071)

Supplementary Figures

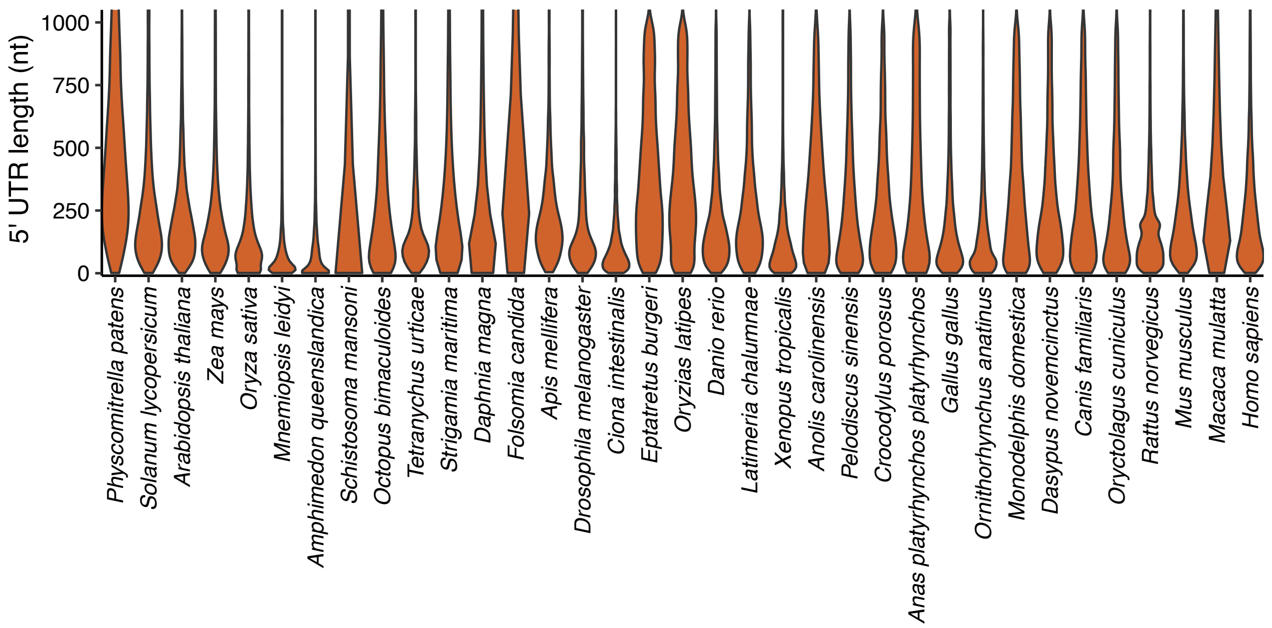

Supplementary Figure 1. The distribution of the 5' untranslated region (UTR) lengths of protein-coding genes with annotated 5' UTRs in each representative species. For a gene with multiple transcript isoforms with a 5' UTR annotation, the longest isoform was used in the analysis. Source data are provided as a Source Data file.

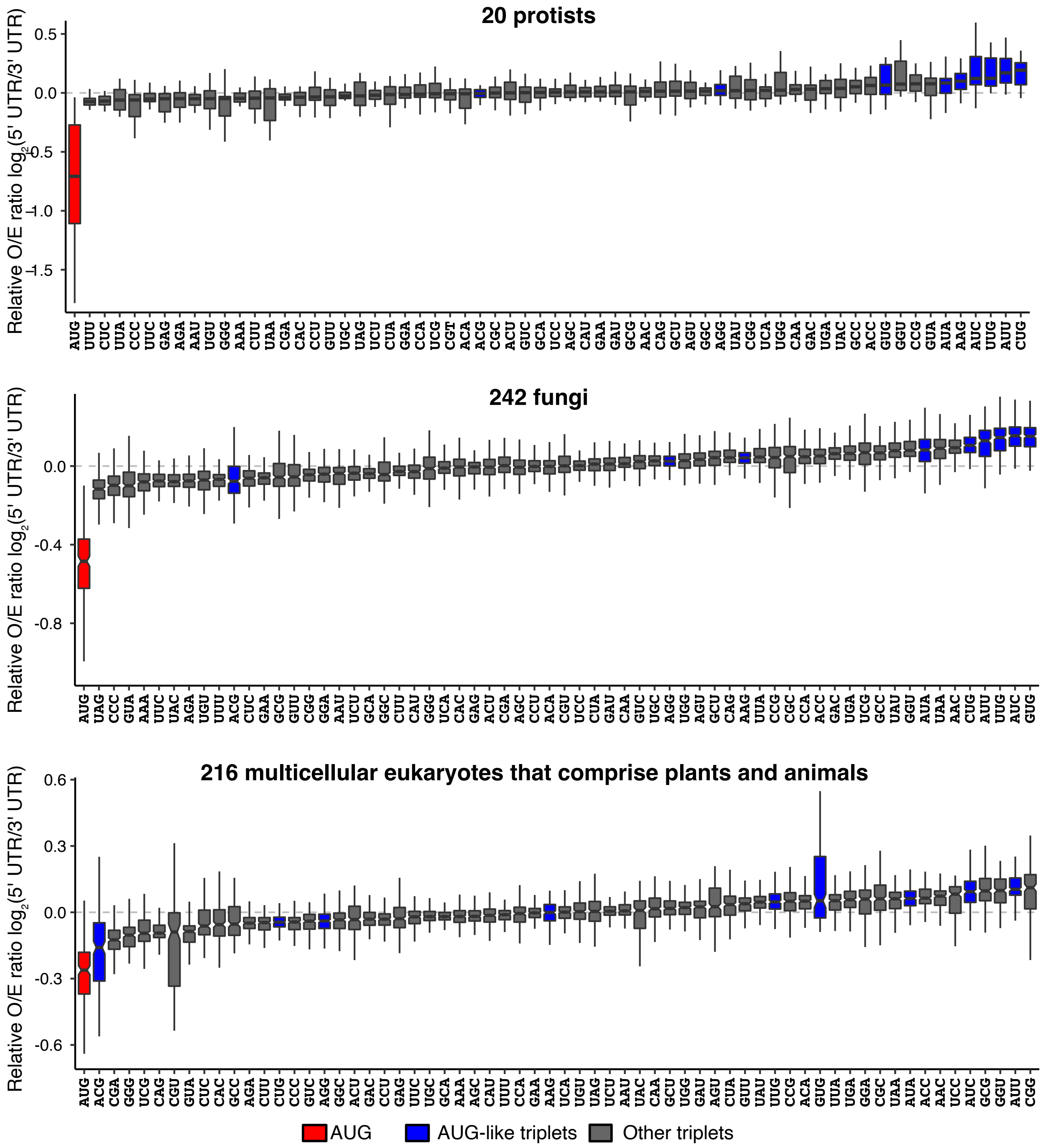

Supplementary Figure 2. The boxplots of relative (5' untranslated region [UTR] versus 3' UTR) ratios of observed over expected numbers(O/E ratio) of all the 64 triplets in 20 protists, 242 fungi, and 216 multicellular plants and animals. Different triplets are ranked by the median values of the relative O/E ratio. The distributions of AUG, AUG-like triplets, and the other triplets are displayed in red, blue, and grey, respectively. Center line, median; box limits, upper and lower quartiles; whiskers, 1.5 times the interquartile range. Source data are provided as a Source Data file.

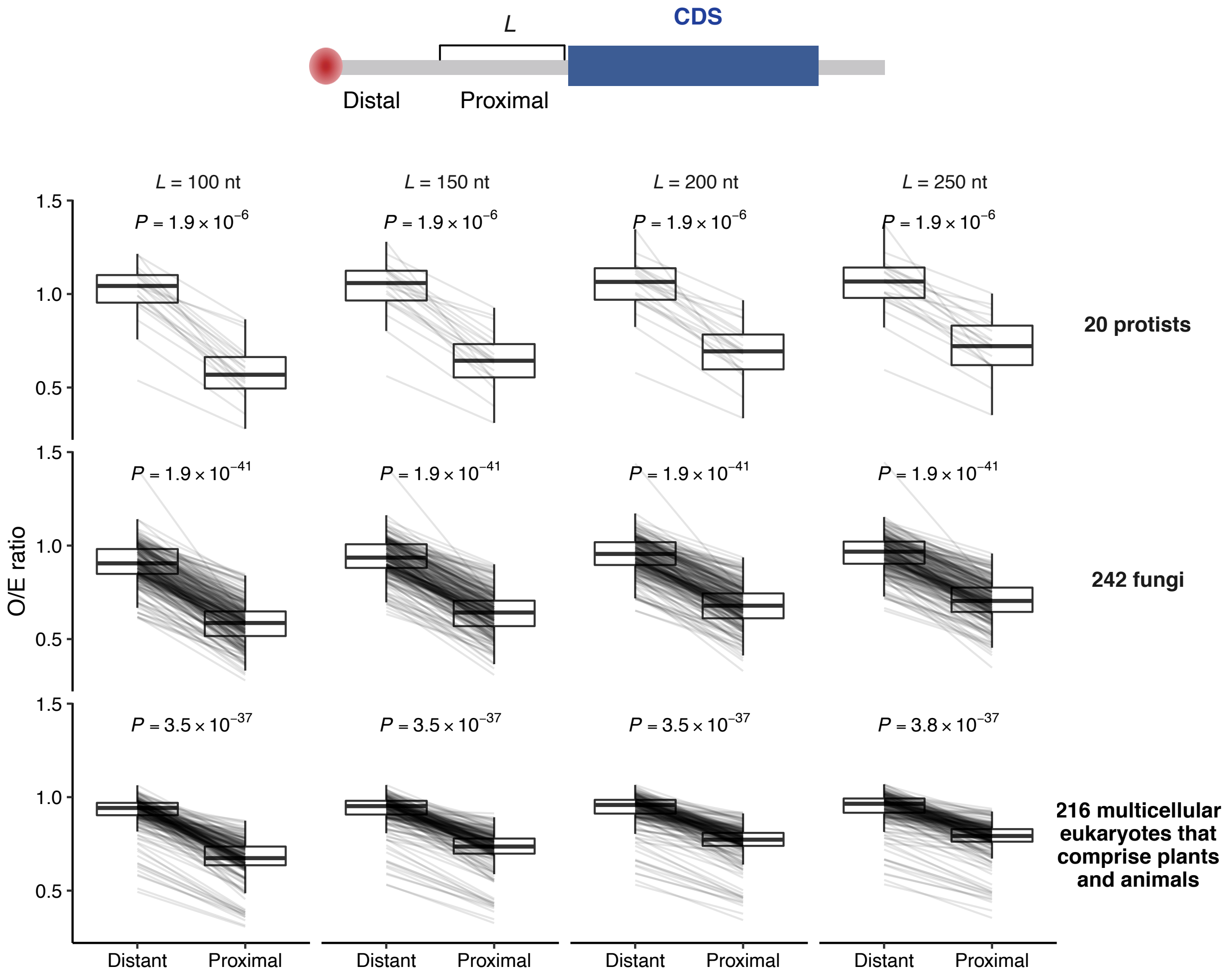

Supplementary Figure 3. Comparison of the ratios of observed over expected number (O/E ratio) of uAUGs that are proximal versus distal to coding sequences (CDSs) in 478 eukaryotes. For each species, the longest transcript of a gene was divided into two parts: proximal (5' untranslated region [UTR] within a distance *L* from the start codon of the CDS) and distal (the remaining 5' UTR region) parts. The number of AUG triplets that are located in the proximal and distal regions were counted. Then, we shuffled the 5' UTR sequences for 1,000 times while maintaining the same di-nucleotide frequency. The median numbers of AUG triplets in the proximal and distal regions in the 1,000 replicates of shuffled sequences were calculated as the expected number of uAUGs in the proximal and distal regions, respectively. Different cutoffs of *L* were used (100 nt, 150 nt, 200 nt, and 250 nt). Two-sided Wilcoxon signed-rank tests were performed to assess the significance of differences. No correction for multiple testing was made since only three tests were performed for each cutoff. Center line, median; box limits, upper and lower quartiles; whiskers, 1.5 times the interquartile range. Source data are provided as a Source Data file.

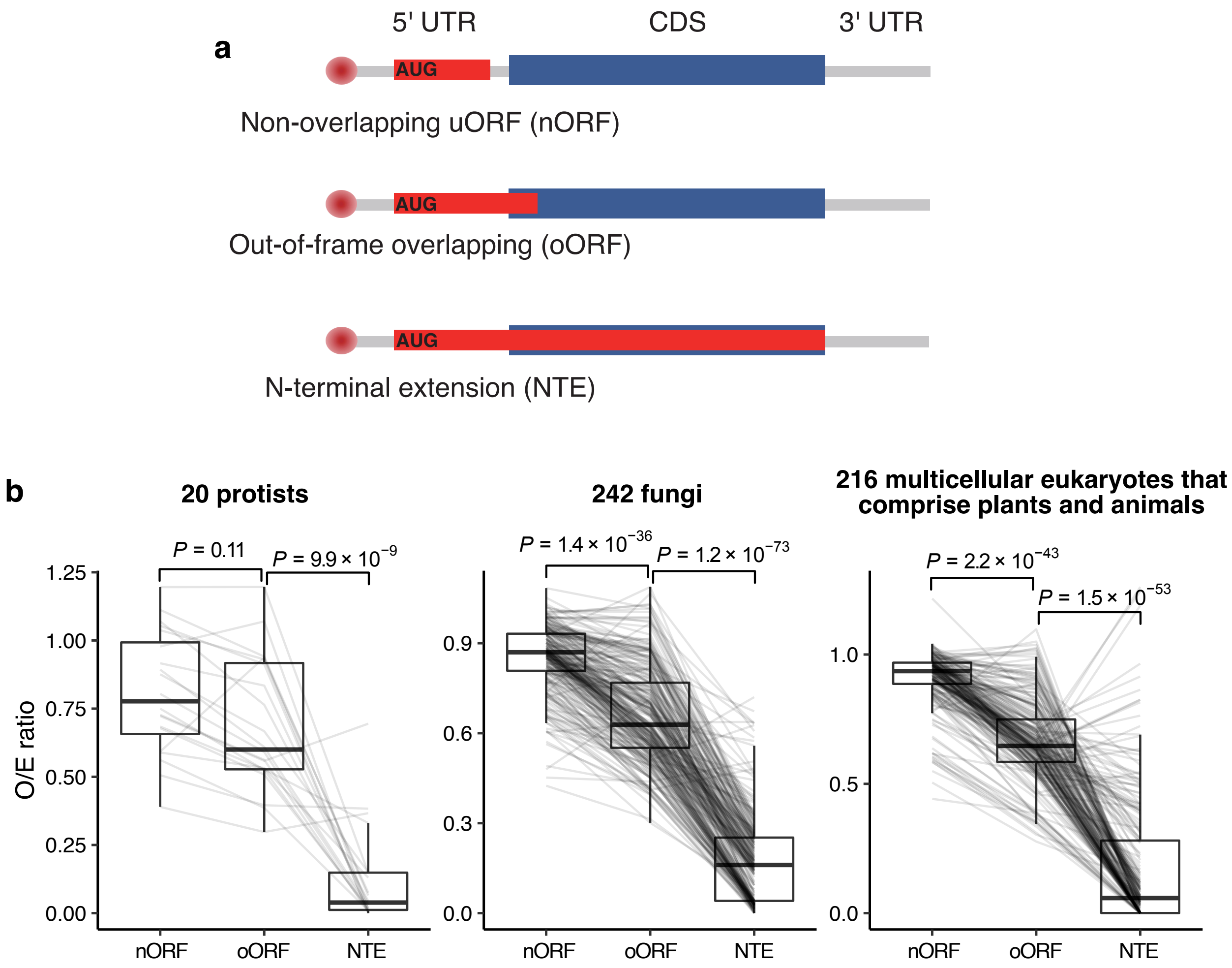

Supplementary Figure 4. The ratios of observed over expected number (O/E ratio) of upstream AUGs (uAUG) of different types in 5' untranslated regions (UTRs).

(**a**) The illustration of different types of uAUGs. Based on the position and frame relative to the downstream coding sequence (CDS), uAUGs can function as the start codons of ORFs in one of the following three categories: (i) nonoverlapping uORFs (nORFs), the stop codons of which are upstream of the start codons of the annotated CDSs; (ii) out-of-frame overlapping uORFs (oORFs), the stop codons of which are located downstream of cAUGs and in different reading frames; and (3) N-terminal extensions (NTEs), which are in fact in-frame overlapping ORFs that share the same stop codon with the annotated CDSs. The details are presented in Ref. [12].

(**b**) The distribution of O/E ratios for different types of uAUGs. The O/E ratio values for the three types of uAUGs of the same species were connected by lines. Two-sided Wilcoxon signed-rank tests were performed to determine the significance of differences. Center line, median; box limits, upper and lower quartiles; whiskers, 1.5 times the interquartile range. Source data are provided as a Source Data file.

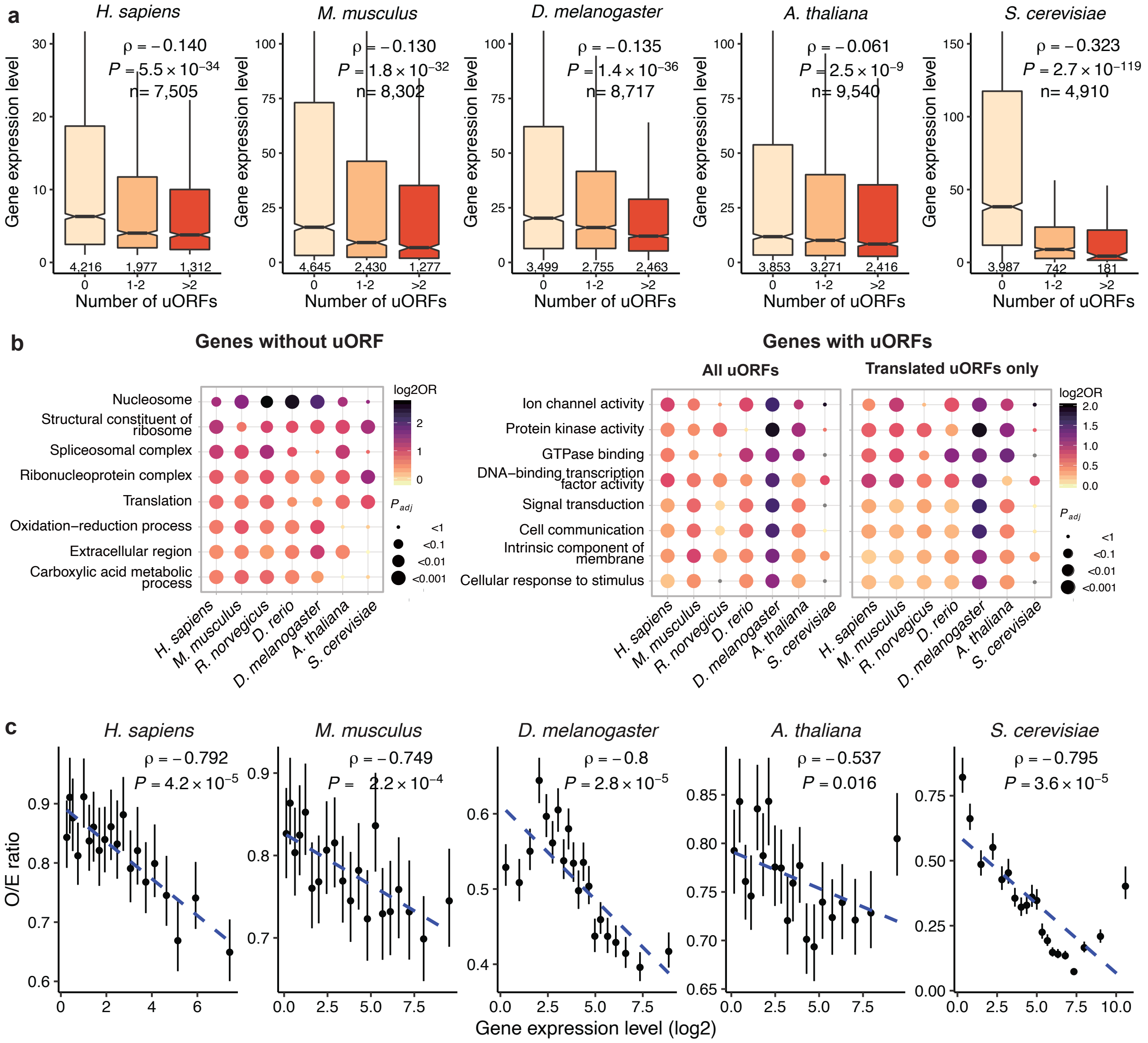

Supplementary Figure 5. Gene expression is one important factor influencing upstream open reading frame (uORF) distribution across genes.

(**a**) The box plots showing the expression levels (log2) of genes with different numbers of uORFs. Spearman's correlations (two-sided) between the expression levels and numbers of uORFs are shown at the top of each panel. For human, mouse, and *A. thaliana*, the abundance of proteins integrated over different tissues[13] was used in the analysis. For fly, the coding sequence (CDS) RPKM values from the ribosome profiling of 12 samples across different developmental stages or tissues and S2 cells [14,15] were averaged for use in the analysis. For yeast, the average protein abundance integrated over different datasets[13] was used. In each dataset, only genes with an expression level (RPKM or ppm) ≥ 1 were analyzed. RPKM, reads per kilobase per million mapped reads; ppm, parts per million, i.e., molecules per million protein molecules in the proteome. Center line, median; box limits, upper and lower quartiles; whiskers, 1.5 times the interquartile range.

(**b**) Gene categories enriched in uORF-free genes (left) and uORF-containing genes (right). In each species, genes belonging to each category were extracted from the annotations provided by Gene Ontology Consortium (Methods). Whether a gene category is enriched in the gene set is assessed with Fisher's exact test. Multiple testing correction was performed with the Benjamini-Hochberg method[16]. The odds ratios (log2) and adjusted *P* values are indicated by the color and size of the points, respectively. For uORF-containing genes, the same

analysis was performed for all the genes containing putative uORFs or only genes containing translated uORFs. Non-redundant representative terms that are significantly enriched in at least five of the seven model organisms were displayed in the plot. See Supplementary Data 3 for the complete list of terms enriched in each species. Some terms are insignificant in yeast, primarily because yeast is a unicellular organism with only 6,600 protein-coding genes, and 955 of those genes contain uORFs, which makes the statistical power of enrichment analyses relatively low in yeast.

(**c**)The relationship between gene expression and the ratio of observed over expected number (O/E ratio) of uORFs. For each species, expressed genes were divided into 20 bins of equal size based on increasing expression levels. The O/E ratio of uORFs was calculated for genes in each bin separately. The median gene expression level of each bin was used in visualization, and the calculation of Spearman's correlation (two-sided). Center point, median O/E ratio in n=1,000 permutation replicates; error bars, the 95% confidence interval of O/E ratios. Source data are provided as a Source Data file.

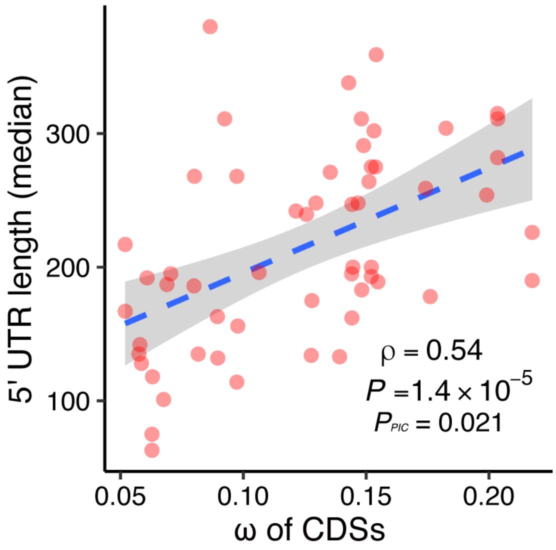

Supplementary Figure 6. Relationship between the genome-wide average number of nonsynonymous changes per nonsynonymous site over the number of synonymous changes per synonymous site (ω) of coding sequences (CDSs) and the median 5' untranslated region (UTR) length among 56 animals. Both Spearman's correlation (two-sided) and the significance of the two-sided phylogenetic independent contrast (PIC) between ω and the median 5' UTR length (P_{PIC}) are shown. The blue line indicates the linear regression fit of the data, and the grey band indicates the standard error of the fit. Source data are provided as a Source Data file.

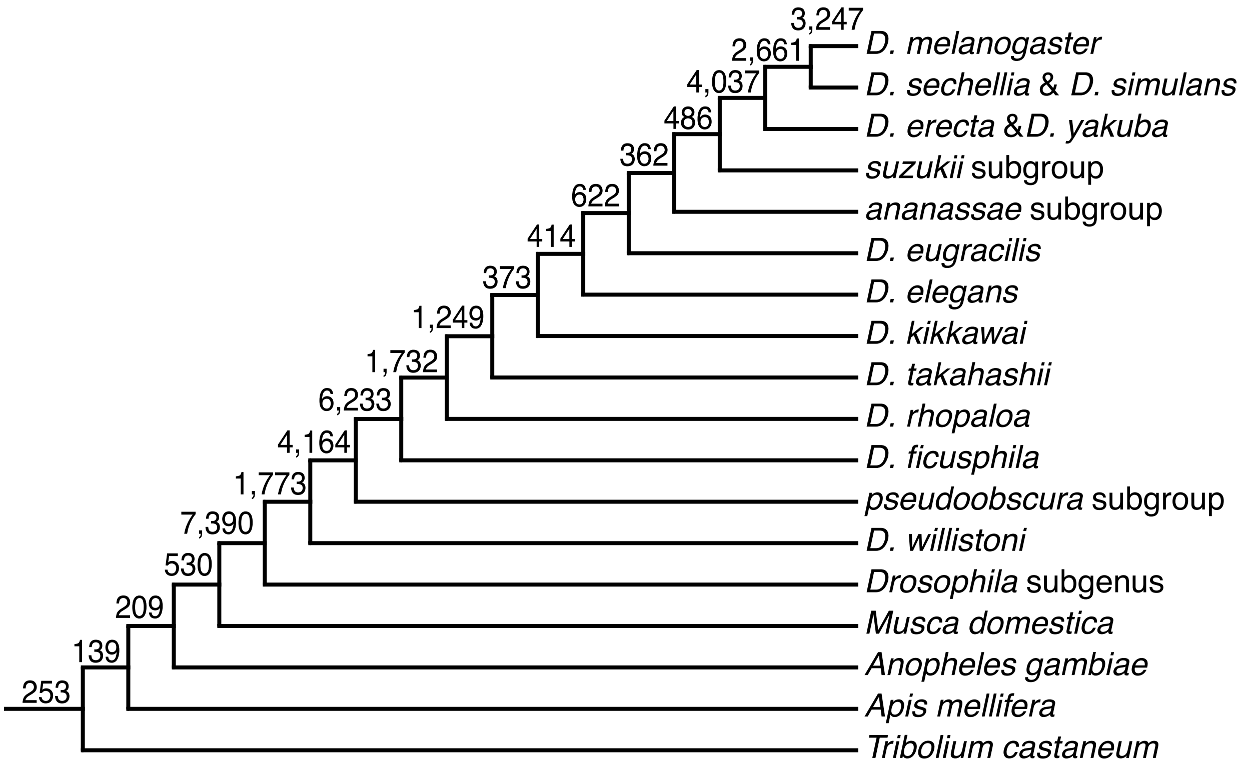

Supplementary Figure 7. The age distribution of upstream open reading frame (uORF) start codons (uoAUGs) in flies. The number of origination events assigned to each branch was based on the most distant clade that has AUG at the orthologuous site for each uoAUG in *D. melanogaster*. Source data are provided as a Source Data file.

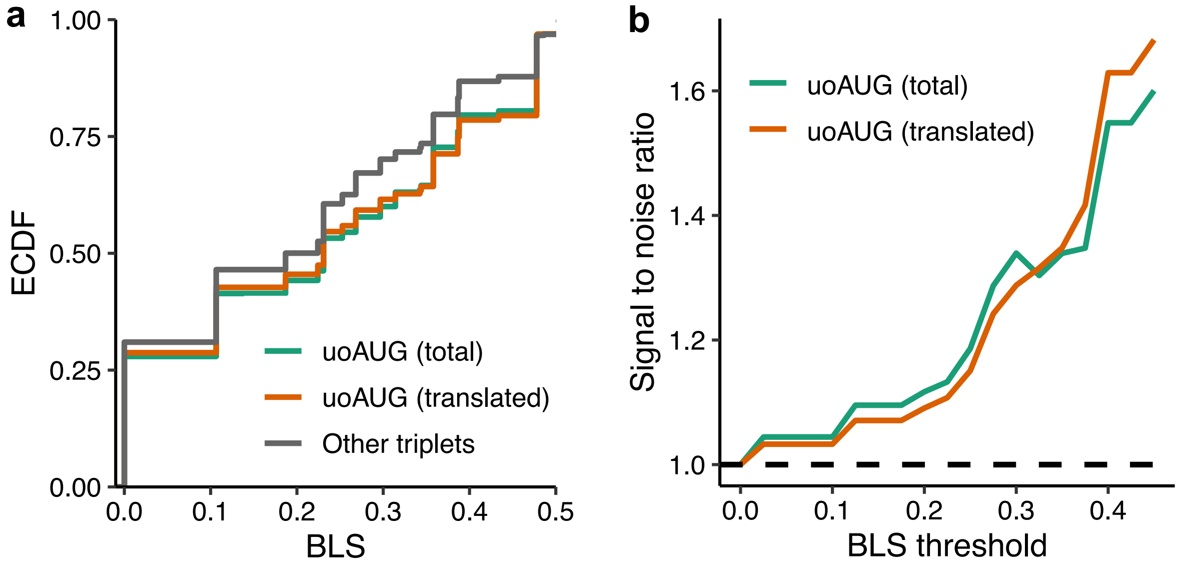

Supplementary Figure 8. The branch length score (BLS) of upstream open reading frame (uORF) start codons (uoAUGs) and other triplets in 5' untranslated regions (UTRs) of yeast.

(**a**) The empirical cumulative distribution frequency (ECDF) of BLS for uoAUGs and the other triplets in 5' UTRs. The BLS of uoAUGs (total or translated) was significantly larger than that of the other triplets (P = 1.5×10^{-13}, 9.5×10^{-11} for total uoAUGs and translated uoAUGs, respectively; two-sided Wilcoxon's rank-sum tests).

(**b**) The signal-to-noise ratio of BLSs for uoAUGs relative to other triplets in 5' UTRs based on different minimum BLS thresholds. Source data are provided as a Source Data file.

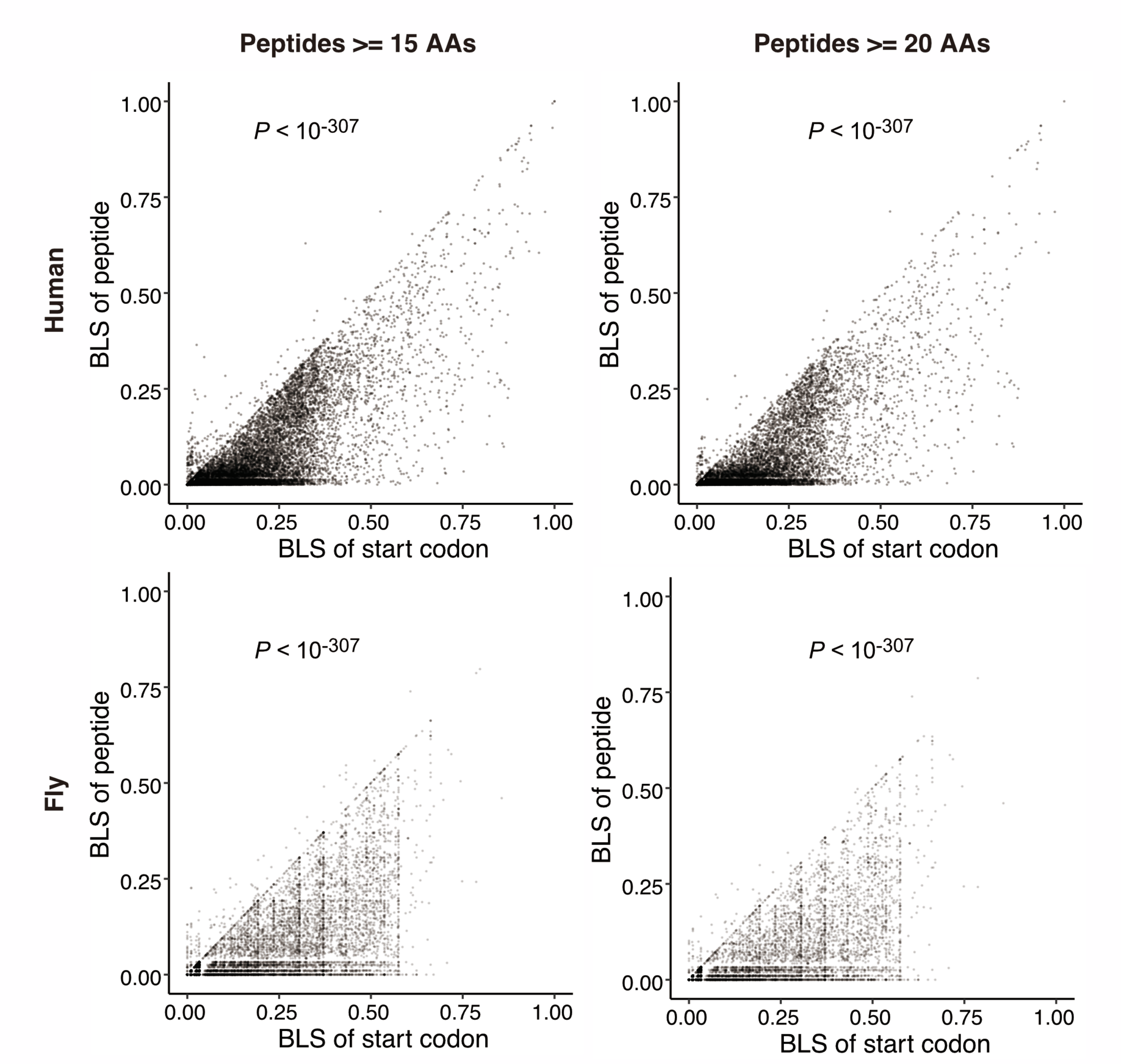

Supplementary Figure 9. The relationship between the branch length scores (BLSs) of upstream open reading frame (uORF) start codons and uORF-encoded peptides in humans and flies. Coding sequences (CDS)-overlapping portions of in-frame overlapping uORFs (oORFs) were excluded. Only uORFs with a minimum peptide length ≥ 15 or 20 amino acids (AAs) were analyzed. Differences in BLS values between uORF start codons and peptides were compared with two-sided Wilcoxon sign-rank tests. Source data are provided as a Source Data file.

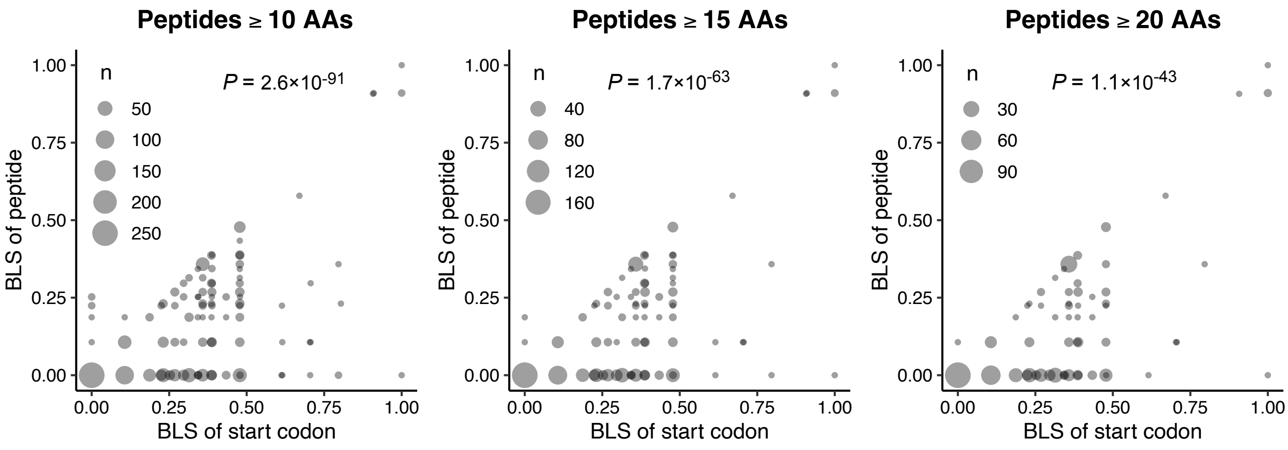

Supplementary Figure 10. The relationship between the branch length scores (BLSs) of upstream open reading frame (uORF) start codons and uORF-encoded peptides in yeasts. Different cutoffs of the minimum peptide length (amino acids, AAs) were used in the analysis. The size of each dot is scaled to the number of uORFs (n). Differences in BLS values between uORF start codons and peptides were compared with two-sided Wilcoxon sign-rank tests. Source data are provided as a Source Data file.

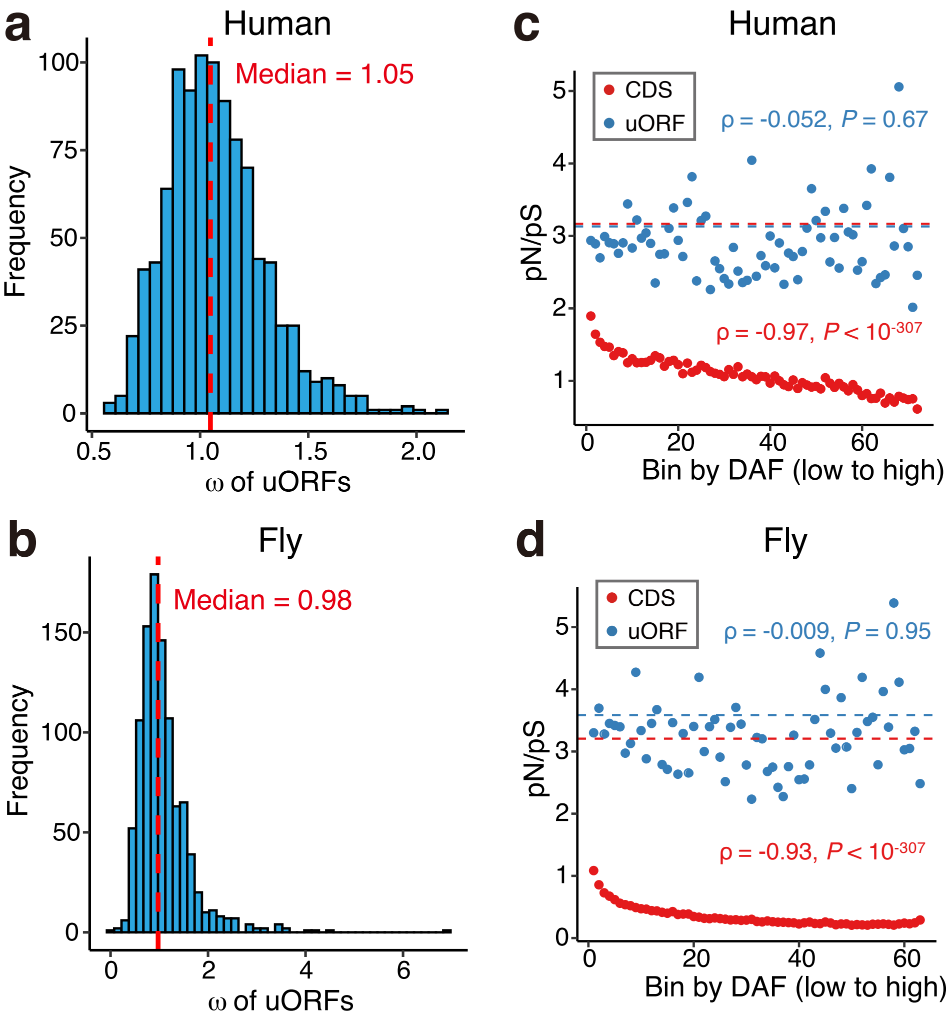

Supplementary Figure 11. Analysis of selective constraints on upstream open reading frames (uORFs) using only translated uORFs.

(**a**) Distribution of the number of nonsynonymous changes per nonsynonymous site over the number of synonymous changes per synonymous site (ω) of human uORFs. Human uORFs were divided into 1,000 bins with equal numbers of uORFs based on increasing start codon Kozak scores. For each bin, the alignments of uORFs between human and rhesus macaque were concatenated to calculate ω. Coding sequence (CDS)-overlapping portions of in-frame overlapping uORFs (oORFs) were excluded from the alignment.

(**b**) Distribution of the ω of fly uORFs. The procedure for ω calculation between fly and *D. simulans* is similar to that in (**a**).

(**c**) The ratio of nonsynonymous to synonymous single-nucleotide polymorphism (SNP) numbers (*pN*/*pS*) of CDSs (red) and uORFs (blue) in bins of increasing derived allele frequency (DAF). SNPs in CDS-overlapping portions of oORFs were excluded. Spearman's correlations (two-sided) between the *pN*/*pS* ratio and the median DAF of each bin for uORFs and CDSs are displayed in the plot.

(**d**) Same as **c** but for fly uORFs. Source data are provided as a Source Data file.

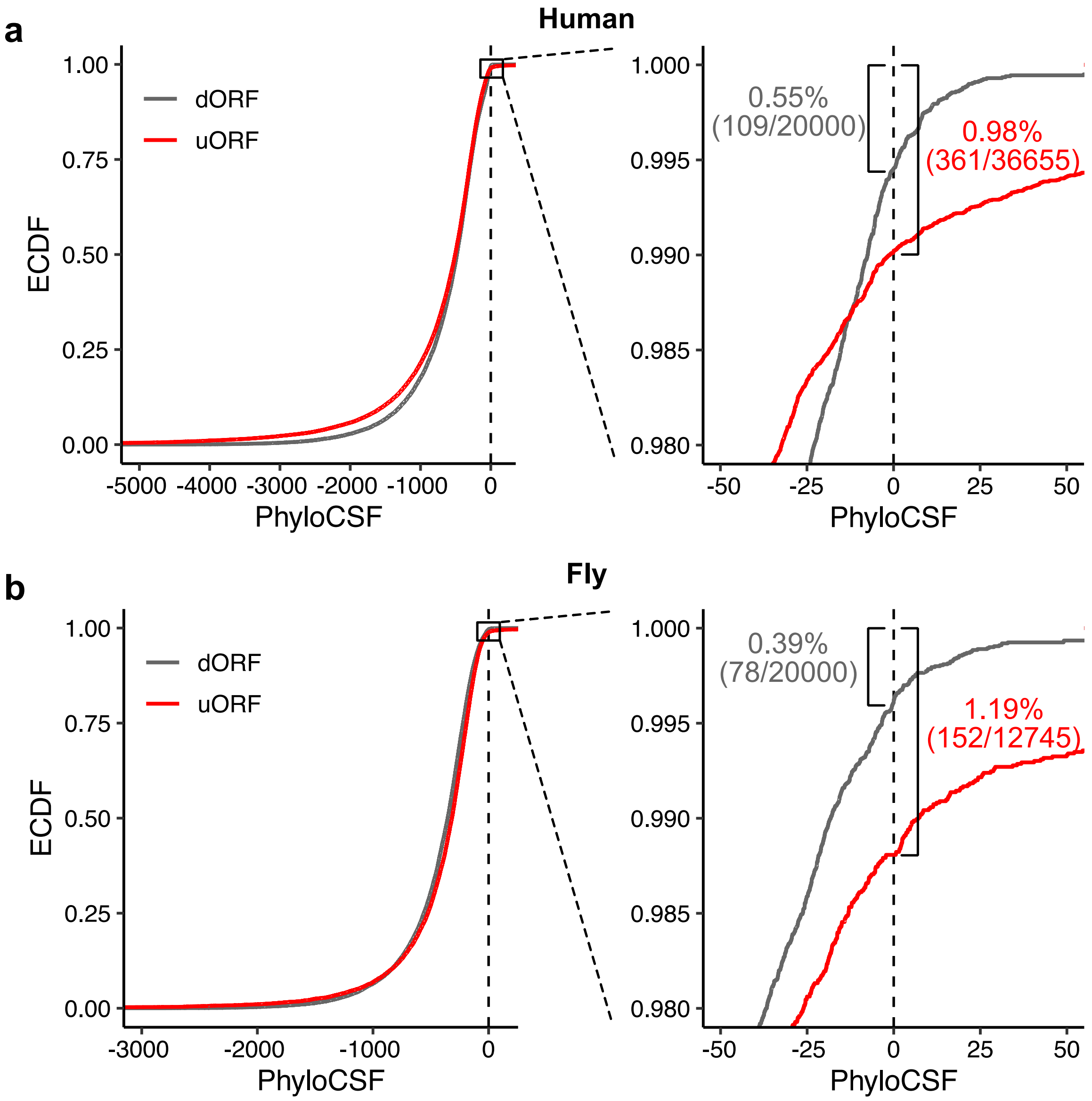

Supplementary Figure 12. The distribution of PhyloCSF of upstream open reading frames (uORFs) and randomly selected downstream ORFs (dORFs) in 3' UTRs.

(**a**) The empirical cumulative distribution function (ECDF) of 36,655 translated uORFs with at least 10 codons in length or 20,000 randomly selected dORFs (⩾10 codons) in humans. The detailed distribution around PhyloCSF = 0 (vertical dashed lines) is shown on the right panel. The fraction of uORFs or dORFs with PhyloCSF > 0 was displayed in the plot.

(**b**) The empirical cumulative distribution function (ECDF) of 12,745 well-translated uORFs with at least 10 codons in length or 20,000 randomly selected dORFs (≥10 codons) in flies. Well-translated uORFs in flies are Classes I-III uORFs, as defined in our previous study[15]. The detailed distribution around PhyloCSF = 0 (vertical dashed lines) is shown on the right panel. The fraction of uORFs or dORFs with PhyloCSF > 0 was displayed in the plot. Source data are provided as a Source Data file.

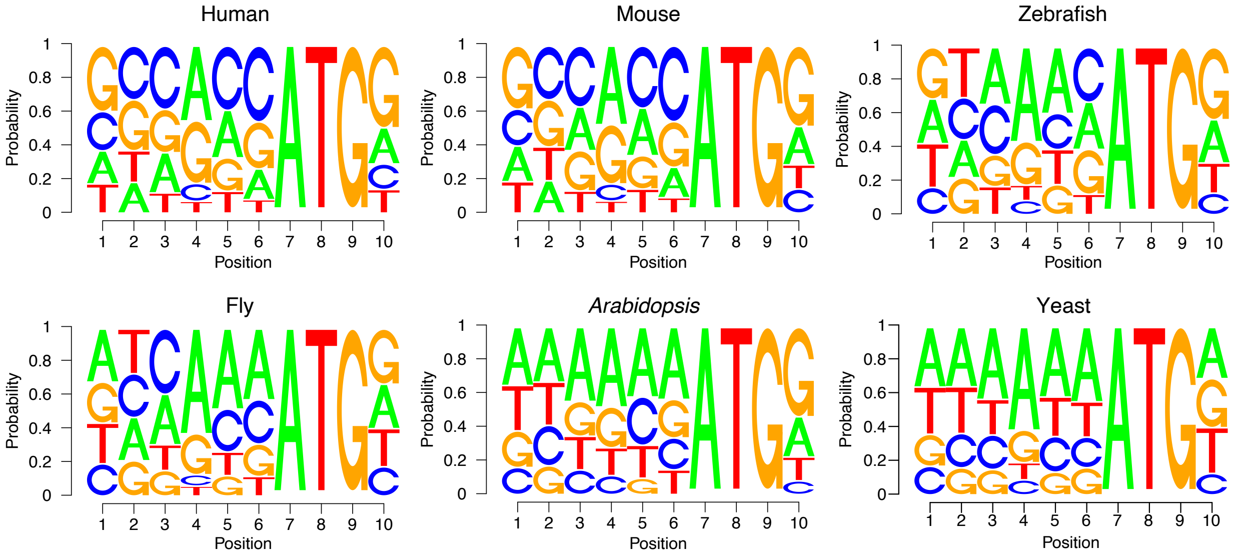

Supplementary Figure 13. The position weight matrixes of coding sequence (CDS) Kozak sequences (PWMK) in several model organisms. For each species, the longest transcripts with 5' untranslated region (UTR) annotation for each gene were used. The frequency of each nucleotide at positions -6 to +4 was tabulated to construct the PWMK. The height of each letter in the plots is scaled to its frequency at the corresponding position. Source data are provided as a Source Data file.

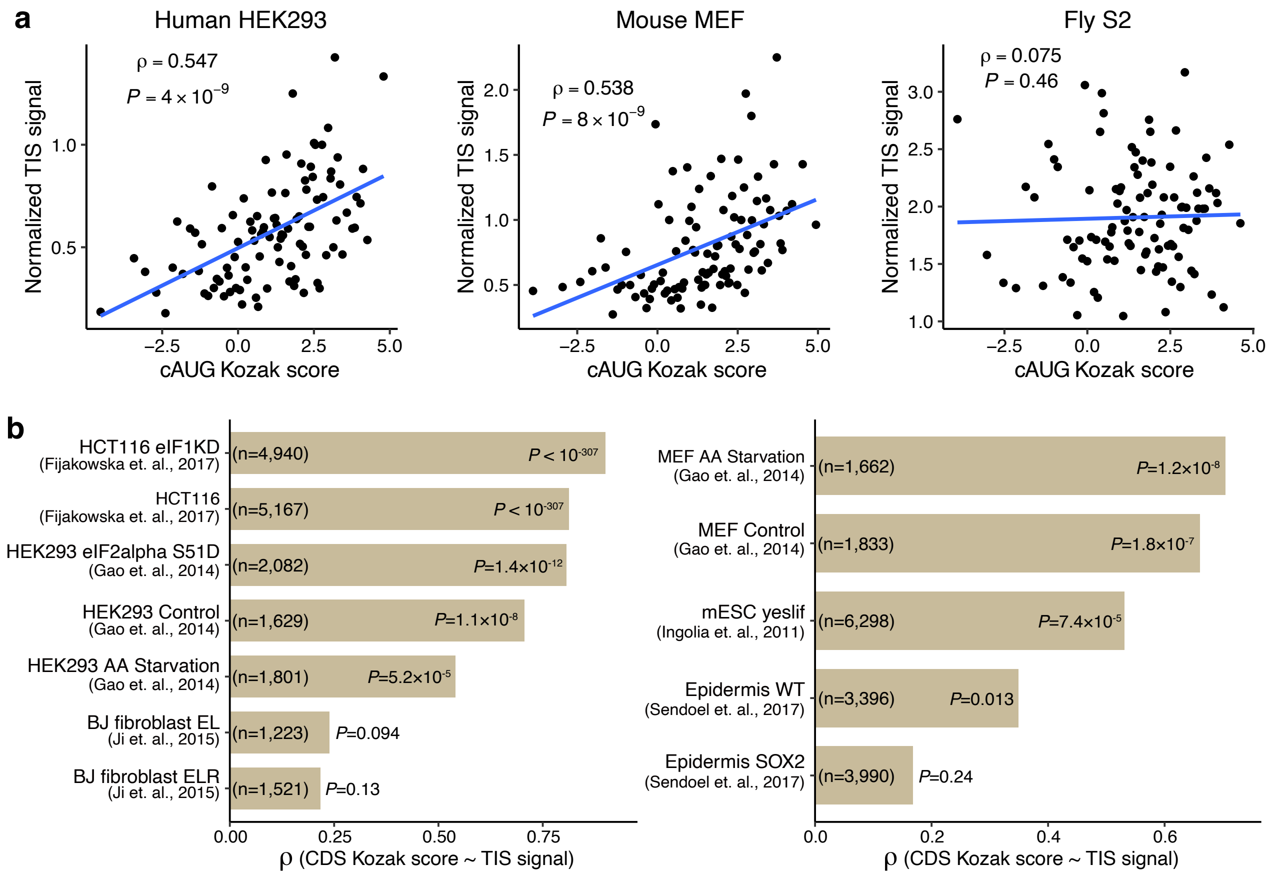

Supplementary Figure 14. The relationship between the Kozak scores and the translational initiation signals of coding sequence (CDS) start codons (cAUGs). Translation initiation site (TIS) profiling and matched RNA-Seq data for human or mouse cell lines and fly S2 cells were obtained from public resources[15,17-22]. For each cAUG in a sample, we calculated the normalized TIS signal by dividing its initiating RPF count with its mean coverage in the matched RNA-Seq data. For each sample, only cAUGs with initiating RPF counts ≥ 2, mRNA counts ≥ 4 (RPF counts ≥5 and mRNA coverage ≥ 10 for S2 cells) were used in the analysis. We ranked cAUGs based on increasing Kozak scores and divided them into 100 bins with equal numbers of cAUGs. The median Kozak score and normalized TIS signal for each bin were used to calculate Spearman's correlations (ρ) and the two-sided *P* values.

(**a**) Scatter plots show the results for HEK293 cells[19], MEF cells[19], and S2 cells[15]. The blue line indicates the linear regression fit.

(**b**) Results for all the human and mouse cell lines. Source data are provided as a Source Data file.

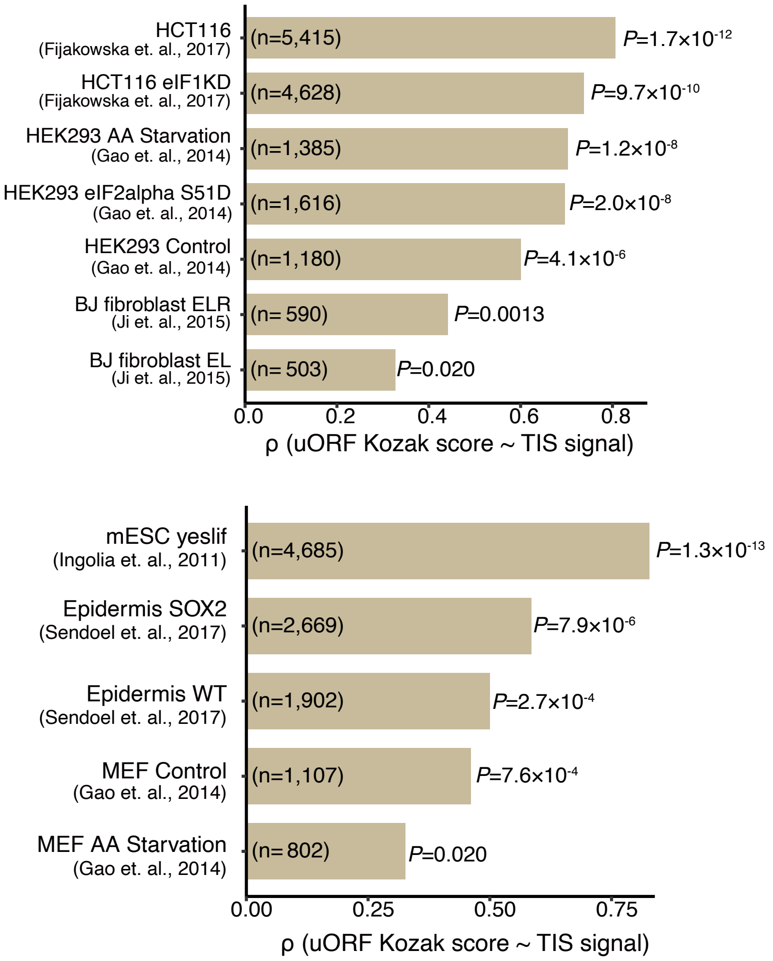

Supplementary Figure 15. The Spearman's correlation between the Kozak scores and the translation initiation signals of upstream open reading frame (uORF) start codons (uoAUGs) in different human (top) or mouse (bottom) cell lines. Translation initiation site (TIS) profiling and matched RNA-Seq data for each sample were obtained from the GWIPs-viz database [17-22]. For each uoAUG in a sample, we calculated the normalized TIS signal by dividing its initiating RPF count with its mean coverage in the matched RNA-Seq data. For each sample, only uoAUGs with initiating RPF counts ≥ 2, mRNA counts ≥ 4 were used in the analysis. We ranked uoAUGs based on increasing Kozak scores and divided them into 50 bins with equal numbers of uoAUGs. The median Kozak score and normalized TIS signal for each bin were used to calculate Spearman's correlations (ρ) and the two-sided *P* values. Source data are provided as a Source Data file.

.

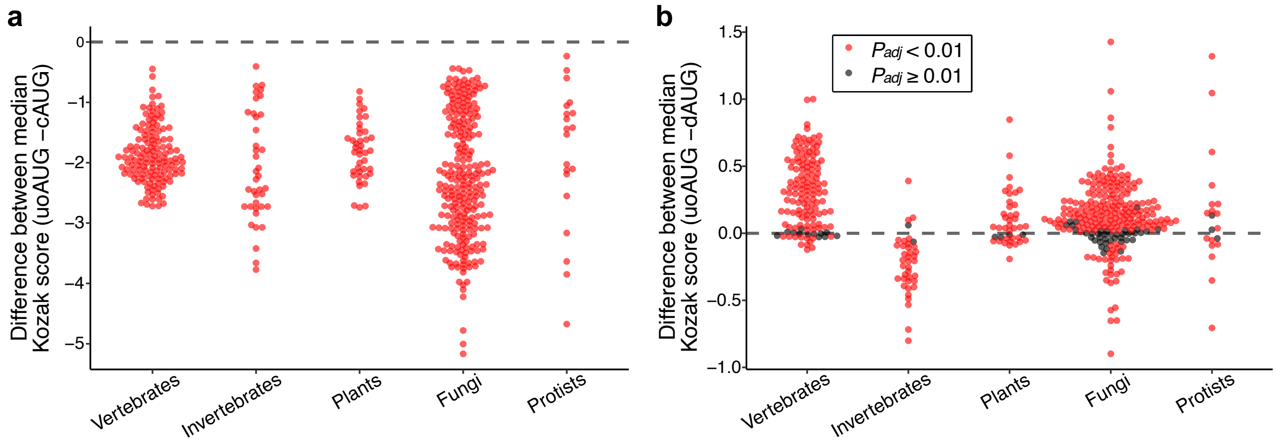

Supplementary Figure 16. Comparisons of Kozak scores of upstream open reading frame (uORF) start codons (uoAUGs), coding sequence (CDS) start codons (cAUGs), and AUGs within 3' UTRs (dAUGs) in 478 eukaryotes.

(**a**) The differences of median Kozak scores between uoAUGs and cAUGs in eukaryotes of different taxa. For each gene in each species, only the longest transcript with 5' UTR annotation was used in the analysis. Two-sided Wilcoxon rank-sum tests were performed to compare the Kozak scores of uoAUGs against cAUGs or dAUGs. *P* values were adjusted for multiple-testing correction.

(**b**) The differences of median Kozak scores between uoAUGs and dAUGs. Source data are provided as a Source Data file.

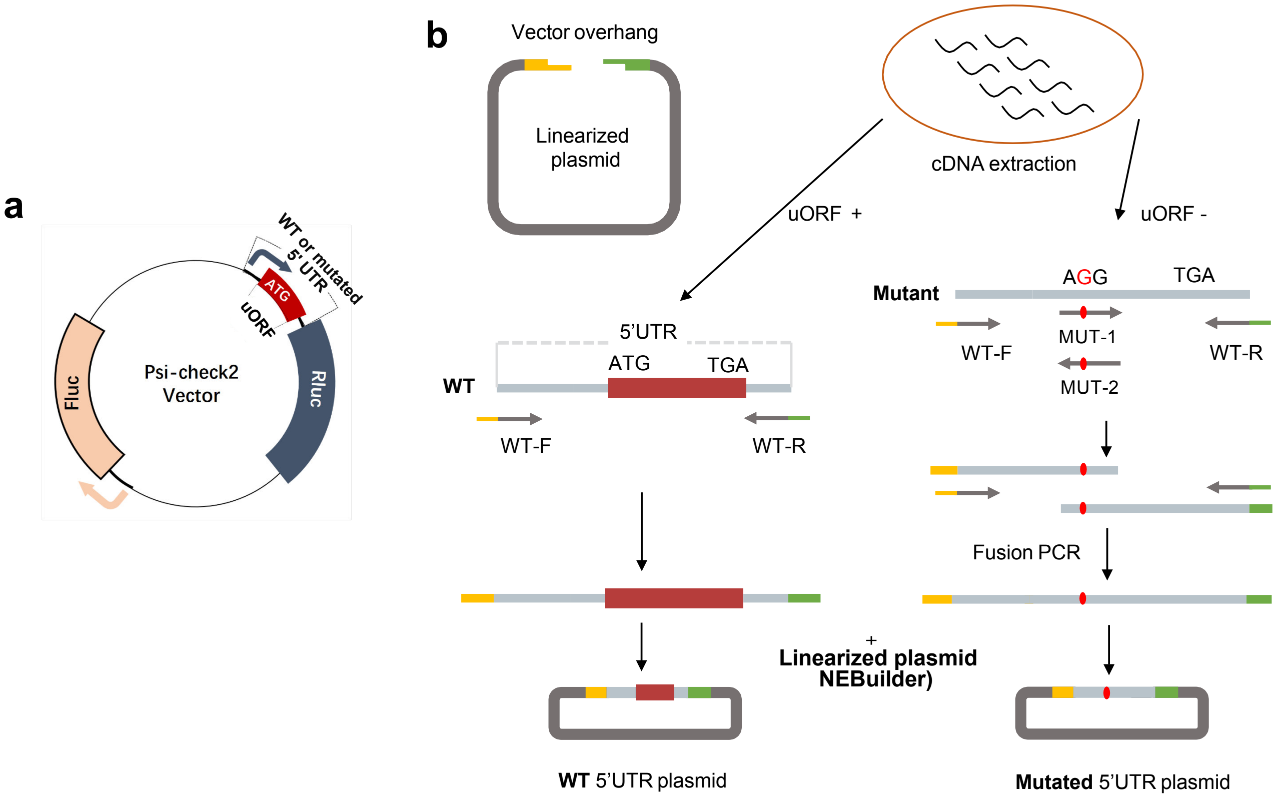

Supplementary Figure 17. The experimental design of dual-luciferase assays.
(**a**) The structure of the Psi-check2 plasmid used for dual-luciferase reporter assays. Wild-type (WT) or mutated 5' untranslated regions (UTRs) were cloned into the upstream region of the Renilla luciferase ORF. The firefly luciferase reporter on the same plasmid was used for internal normalization.
(**b**) The scheme of plasmid construction for dual-luciferase reporter assays. The WT fragment was obtained directly by PCR from cDNAs. The mutant fragment was obtained by fusion PCR. If the position of an upstream open reading frame (uORF) variant is close to the 5' or 3' end of the 5' UTR, the mutated fragment was obtained by normal PCR as performed for the WT fragment but with mutated primers. The WT or mutated fragment is ligated with the linearized reporter plasmid to get the final plasmid.

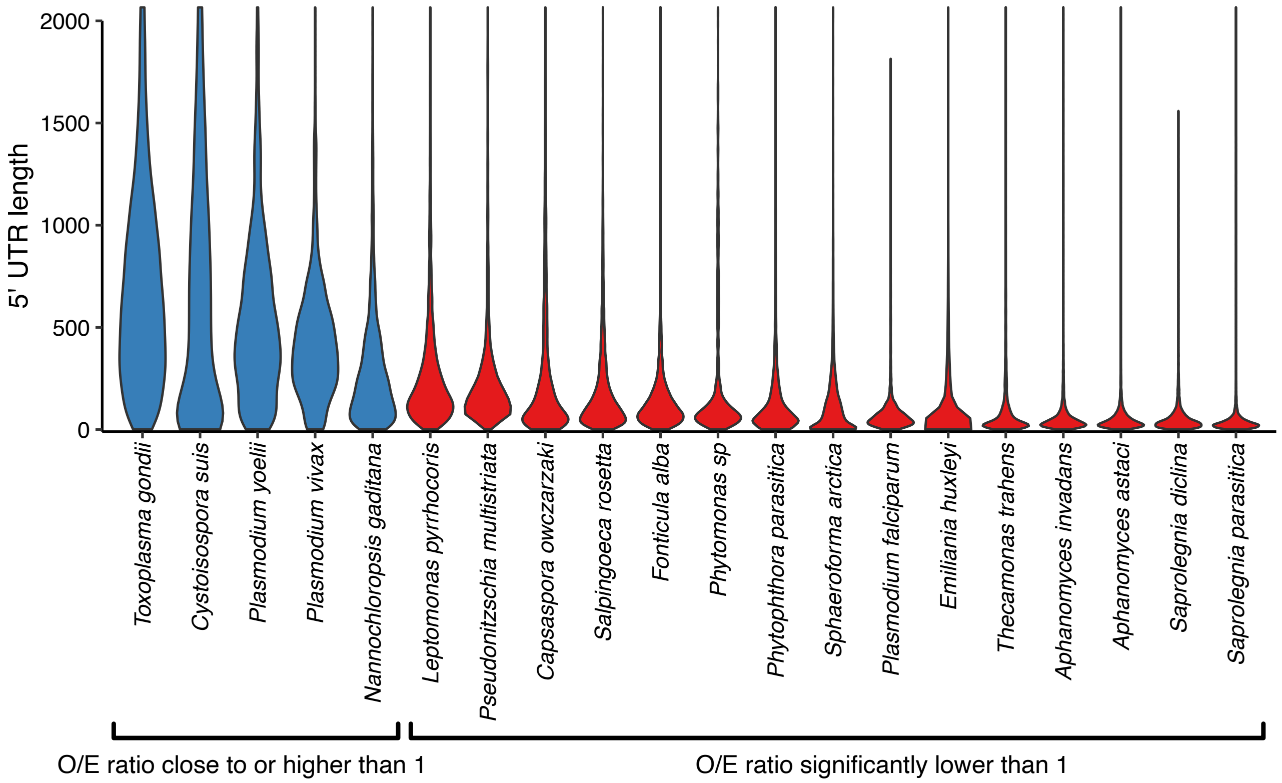

Supplementary Figure 18. The distribution of the 5' untranslated region (UTR) lengths of protein-coding genes with annotated 5' UTRs in 20 protists. For a gene with multiple transcript isoforms with a 5' UTR annotation, the longest isoform was used in the analysis. Different species are ranked by the median lengths of annotated 5' UTRs in descending order. The distribution for the 15 protists with ratios of observed over expected number (O/E ratio) of AUG triplets in 5' UTRs significantly lower than 1 were shown in red, while the remaining 5 protists in blue. Source data are provided as a Source Data file.

Supplementary References

1 Ruokonen, M., Aarvak, T. & Madsen, J. Colonization history of the high-arctic pink-footed goose Anser brachyrhynchus. *Mol Ecol* **14**, 171-178, doi:10.1111/j.1365-294X.2004.02380.x (2005).

2 MacEachern, S., Hayes, B., McEwan, J. & Goddard, M. An examination of positive selection and changing effective population size in Angus and Holstein cattle populations (Bos taurus) using a high density SNP genotyping platform and the contribution of ancient polymorphism to genomic diversity in Domestic cattle. *BMC Genomics* **10**, 181, doi:10.1186/1471-2164-10-181 (2009).

3 Clark, N. A. *et al.* First formal estimate of the world population of the Critically Endangered spoon-billed sandpiper Calidris pygmaea. *Oryx* **52**, 137-146, doi:10.1017/S0030605316000806 (2018).

4 Freedman, A. H. *et al.* Genome sequencing highlights the dynamic early history of dogs. *PLoS Genet* **10**, e1004016, doi:10.1371/journal.pgen.1004016 (2014).

5 Schmitz, J. *et al.* Genome sequence of the basal haplorrhine primate Tarsius syrichta reveals unusual insertions. *Nat Commun* **7**, 12997, doi:10.1038/ncomms12997 (2016).

6 Gossmann, T. I., Keightley, P. D. & Eyre-Walker, A. The effect of variation in the effective population size on the rate of adaptive molecular evolution in eukaryotes. *Genome Biol Evol* **4**, 658-667, doi:10.1093/gbe/evs027 (2012).

7 Xue, C. *et al.* The population genomics of rhesus macaques (Macaca mulatta) based on whole-genome sequences. *Genome Res* **26**, 1651-1662, doi:10.1101/gr.204255.116 (2016).

8 Salcedo, T., Geraldes, A. & Nachman, M. W. Nucleotide variation in wild and inbred mice. *Genetics* **177**, 2277-2291, doi:10.1534/genetics.107.079988 (2007).

9 Yu, N., Jensen-Seaman, M. I., Chemnick, L., Ryder, O. & Li, W. H. Nucleotide diversity in gorillas. *Genetics* **166**, 1375-1383 (2004).

10 Mailund, T., Dutheil, J. Y., Hobolth, A., Lunter, G. & Schierup, M. H. Estimating divergence time and ancestral effective population size of Bornean and Sumatran orangutan subspecies using a coalescent hidden Markov model. *PLoS Genet* **7**, e1001319, doi:10.1371/journal.pgen.1001319 (2011).

11 Deinum, E. E. *et al.* Recent Evolution in Rattus norvegicus Is Shaped by Declining Effective Population Size. *Mol Biol Evol* **32**, 2547-2558, doi:10.1093/molbev/msv126 (2015).

12 Zhang, H., Wang, Y. & Lu, J. Function and Evolution of Upstream ORFs in Eukaryotes. *Trends in Biochemical Sciences* **44**, 782-794, doi:10.1016/j.tibs.2019.03.002 (2019).

13 Wang, M., Herrmann, C. J., Simonovic, M., Szklarczyk, D. & von Mering, C. Version 4.0 of PaxDb: Protein abundance data, integrated across model organisms, tissues, and cell-lines. *Proteomics* **15**, 3163-3168, doi:10.1002/pmic.201400441 (2015).

14 Kronja, I. *et al.* Widespread changes in the posttranscriptional landscape at the Drosophila oocyte-to-embryo transition. *Cell Rep* **7**, 1495-1508, doi:10.1016/j.celrep.2014.05.002 (2014).

15 Zhang, H. *et al.* Genome-wide maps of ribosomal occupancy provide insights into adaptive evolution and regulatory roles of uORFs during Drosophila development. *PLoS Biol* **16**, e2003903, doi:10.1371/journal.pbio.2003903 (2018).

16 Benjamini, Y. & Hochberg, Y. Controlling the false discovery rate: a practical and powerful approach to multiple testing. *Journal of the Royal statistical society: series B (Methodological)* **57**, 289-300 (1995).

17 Michel, A. M. *et al.* GWIPS-viz: development of a ribo-seq genome browser. *Nucleic Acids Res* **42**, D859-864, doi:10.1093/nar/gkt1035 (2014).

18 Ingolia, N. T., Lareau, L. F. & Weissman, J. S. Ribosome profiling of mouse embryonic stem cells reveals the complexity and dynamics of mammalian proteomes. *Cell* **147**, 789-802, doi:10.1016/j.cell.2011.10.002 (2011).

19 Gao, X. *et al.* Quantitative profiling of initiating ribosomes in vivo. *Nat Methods* **12**, 147-153, doi:10.1038/nmeth.3208 (2015).

20 Ji, Z., Song, R., Regev, A. & Struhl, K. Many lncRNAs, 5'UTRs, and pseudogenes are translated and some are likely to express functional proteins. *Elife* **4**, e08890, doi:10.7554/eLife.08890 (2015).

21 Fijalkowska, D. *et al.* eIF1 modulates the recognition of suboptimal translation initiation sites and steers gene expression via uORFs. *Nucleic Acids Res* **45**, 7997-8013, doi:10.1093/nar/gkx469 (2017).

22 Sendoel, A. *et al.* Translation from unconventional 5' start sites drives tumour initiation. *Nature* **541**, 494-499, doi:10.1038/nature21036 (2017).
