# Supplementary material for: Determinants of genome-wide distribution and evolution of uORFs in eukaryotes
Source: Nat Commun. 2021 Feb 17;12:1076. doi: 10.1038/s41467-021-21394-y (PMC7889888; doi:10.1038/s41467-021-21394-y)
Supplement: Supplementary file 3 — Description of Additional Supplementary Files [file 41467_2021_21394_MOESM3_ESM.pdf]

Description of Additional Supplementary Files

Supplementary Data 1.

The summary of protein-coding genes and AUG triplets in 5' untranslated regions (uAUGs) in 478 eukaryotes.

Supplementary Data 2.

The Spearman's correlations between gene expression level and the number of upstream open reading frames (uORFs) in 5' untranslated regions (UTRs) across different tissues, developmental stages, or cell lines in representative model organisms.

Supplementary Data 3.

The complete list of significant terms (adjusted $P < 0.1$) for gene ontology analyses of genes with or without upstream open reading frames (uORFs) in representative model organisms as presented in Supplementary Fig. 5b.

Supplementary Data 4.

The accession numbers and corresponding sample names of public Mass Spectrometry datasets analyzed in this study.

Supplementary Data 5.

The list of upstream open reading frames (uORFs) detected in Mass Spectrometry datasets.

Supplementary Data 6.

The Position Weight Matrices of Kozak sequence context around coding sequence (CDS) start codons in 478 eukaryotes.

Supplementary Data 7.

The names and sequences of all the primers used in molecular cloning of wild type and mutated 5' untranslated regions (UTRs) for luciferase reporter assays
