# Supplementary material for: Determinants of genome-wide distribution and evolution of uORFs in eukaryotes
Source: Nat Commun. 2021 Feb 17;12:1076. doi: 10.1038/s41467-021-21394-y (PMC7889888; doi:10.1038/s41467-021-21394-y)
Supplement: Supplementary file 11 — Reporting Summary [file 41467_2021_21394_MOESM11_ESM.pdf]

nature research

Corresponding author(s): Jian Lu

Last updated by author(s): Dec 20, 2020

Reporting Summary

Nature Research wishes to improve the reproducibility of the work that we publish. This form provides structure for consistency and transparency in reporting. For further information on Nature Research policies, see our Editorial Policies and the Editorial Policy Checklist.

Statistics

For all statistical analyses, confirm that the following items are present in the figure legend, table legend, main text, or Methods section.

n/a	Confirmed	
☐	☒	The exact sample size (*n*) for each experimental group/condition, given as a discrete number and unit of measurement
☐	☒	A statement on whether measurements were taken from distinct samples or whether the same sample was measured repeatedly
☐	☒	The statistical test(s) used AND whether they are one- or two-sided<br>*Only common tests should be described solely by name; describe more complex techniques in the Methods section.*
☐	☒	A description of all covariates tested
☐	☒	A description of any assumptions or corrections, such as tests of normality and adjustment for multiple comparisons
☐	☒	A full description of the statistical parameters including central tendency (e.g. means) or other basic estimates (e.g. regression coefficient) AND variation (e.g. standard deviation) or associated estimates of uncertainty (e.g. confidence intervals)
☐	☒	For null hypothesis testing, the test statistic (e.g. *F*, *t*, *r*) with confidence intervals, effect sizes, degrees of freedom and *P* value noted<br>*Give P values as exact values whenever suitable.*
☒	☐	For Bayesian analysis, information on the choice of priors and Markov chain Monte Carlo settings
☒	☐	For hierarchical and complex designs, identification of the appropriate level for tests and full reporting of outcomes
☐	☒	Estimates of effect sizes (e.g. Cohen's *d*, Pearson's *r*), indicating how they were calculated

Our web collection on statistics for biologists contains articles on many of the points above.

Software and code

Policy information about availability of computer code

Data collection	No software was used for data collection.
Data analysis	The data investigated in this study were mostly analyzed using R statistical software (v3.6). The following software was used in this study: uShuffle v1.1; BLAST+ v2.6.0; MUSCLE v2.8.31; EMBOSS v6.6.0; PAML 4.7b; BUSCO v3; python v3.7.4; biopython v1.74; AsymptoticMK (version number unavailable); bigWigAverageOverBed v2; clusterProfiler v3.12.0; topGO v2.36.0; ete3 v3.1.1; ape v5.3; exonerate v2.2; SnpEff v4.3t; PhyloCSF v20121028; MaxQuant v1.6.5; OpenMS v2.3.0; pFind v3;PeptideMatch v1.0; TFBSTools v1.22.0; STAR v2.4.0j; htseq-count v0.11.0; DESeq2 v1.24.0; bedtools v2.27.1; data.table v1.12.2; ggplot2 v3.3.2. The custom scripts used in this study are available from figshare (doi: 10.6084/m9.figshare.12612068).

For manuscripts utilizing custom algorithms or software that are central to the research but not yet described in published literature, software must be made available to editors and reviewers. We strongly encourage code deposition in a community repository (e.g. GitHub). See the Nature Research guidelines for submitting code & software for further information.

Data

Policy information about availability of data

All manuscripts must include a data availability statement. This statement should provide the following information, where applicable:

- Accession codes, unique identifiers, or web links for publicly available datasets
- A list of figures that have associated raw data
- A description of any restrictions on data availability

The putative uORFs and NTEs annotated in this study are available from figshare (https://doi.org/10.6084/m9.figshare.9980441.v4). The following public data were analyzed in this study: 1) gene annotations, cDNA sequences and genome sequences from Ensembl Genome Browser (https://www.ensembl.org and http://ensemblgenomes.org); 2) the transcript ends of yeast mRNAs from Gene Expression Omnibus (GEO, https://www.ncbi.nlm.nih.gov/geo) under accession number

GSE490261; 3) functional annotation of gene categories from The Gene Ontology Resource(http://geneontology.org); 4) gene expression data in model organisms from previous studies as listed in Supplementary Data 2; 5) Ribo-Seq data from GWIPs-viz database (https://gwips.ucc.ie) and our previous study32; 6) the effective population size reported in previous studies as listed in Supplementary Table 2; 7) single nucleotide polymorphisms from the 1000 Genomes Project (https://www.internationalgenome.org/data) and DGRP2 (http://dgrp2.gnets.ncsu.edu); 8) Multiple genome alignments from UCSC Genome Browser (https://genome.ucsc.edu); 9) Annotation of potential functional uORFs from McGillivray et al.17; 10) mass spectrometry datasets from ProteomeCentral (http://proteomecentral.proteomexchange.org) as listed in Supplementary Data 4; 11) RNA-Seq and Ribo-Seq of human lymphoblastoid cell lines from the Gilad/Pritchard group (http://eqtl.uchicago.edu/RNA_Seq_data and GSE61742 from GEO). Source Data are provided with this paper and deposited in figshare as well (https://doi.org/10.6084/m9.figshare.12612068.v2).

Field-specific reporting

Please select the one below that is the best fit for your research. If you are not sure, read the appropriate sections before making your selection.

- [x] Life sciences
- [ ] Behavioural & social sciences
- [ ] Ecological, evolutionary & environmental sciences

For a reference copy of the document with all sections, see nature.com/documents/nr-reporting-summary-flat.pdf

Life sciences study design

All studies must disclose on these points even when the disclosure is negative.

Sample size	No statistical methods were used to predetermine the sample size. All the available genome annotation of eukaryotes from Ensembl Genome Browser were obtained. To ensure reproducibility while saving time, about 40 variants were randomly chosen for experimental validation for both canonical and noncanonical uORFs.
Data exclusions	Species in which less than 25% protein-coding genes have annotated 5' UTR were excluded since the incomplete 5' UTR annotation would preclude the reliable estimation of the degree of uAUG depletion in a species. Among the species meet this criteria, Ichthyophthirius multifiliis was excluded since UAA and UAG are reassigned to encode glutamine in this species, which would interfere with the uORF and NTE prediction. The exclusion criteria were not pre-established in previous studies.
Replication	5 independent biological repetitions were performed for the luciferase reporter assay for both the uORF and non-uORF alleles of the 80 variants. For 15 of the 2x80=160 assays, 4 of the 5 repetitions were successful. For the remaining 145 assays, all the five repetitions were successful. No other experiments were performed in this study.
Randomization	Not relevant to this study since no group allocation was involved.
Blinding	Not relevant to this study since no group allocation was involved.

Reporting for specific materials, systems and methods

We require information from authors about some types of materials, experimental systems and methods used in many studies. Here, indicate whether each material, system or method listed is relevant to your study. If you are not sure if a list item applies to your research, read the appropriate section before selecting a response.

Materials & experimental systems

n/a	Involved in the study	
x		Antibodies
	x	Eukaryotic cell lines
x		Palaeontology and archaeology
x		Animals and other organisms
x		Human research participants
x		Clinical data
x		Dual use research of concern

Methods

n/a	Involved in the study	
x		ChIP-seq
x		Flow cytometry
x		MRI-based neuroimaging

Eukaryotic cell lines

Policy information about cell lines

Cell line source(s)	HEK293FT cells were purchased from the Cell Bank of the Chinese Academy of Sciences.
Authentication	No authentication was performed.
Mycoplasma contamination	TransSafeTM Mycoplasma Elimination Reagent (TransMyco-3) was used to remove mycoplasma contamination before experiments. The cell lines used for formal experiments were tested negative for mycoplasma contamination.
Commonly misidentified lines (See ICLAC register)	None of the commonly misidentified lines was used in this study.
